# The Impact of Family Therapy Participation on Youths and Young Adult Engagement and Retention in a Telehealth Intensive Outpatient Program: Quality Improvement Analysis

**DOI:** 10.2196/45305

**Published:** 2023-04-20

**Authors:** Katie R Berry, Kate Gliske, Clare Schmidt, Jaime Ballard, Michael Killian, Caroline Fenkel

**Affiliations:** 1 Charlie Health, Inc Bozeman, MT United States; 2 University of Minnesota St. Paul, MN United States; 3 College of Social Work Florida State University Tallahassee, FL United States

**Keywords:** adolescents, family therapy, intensive outpatient, mental health, treatment engagement, young adults

## Abstract

**Background:**

Early treatment dropout among youths and young adults (28%-75%) puts them at risk for poorer outcomes. Family engagement in treatment is linked to lower dropout and better attendance in outpatient, in-person treatment. However, this has not been studied in intensive or telehealth settings.

**Objective:**

We aimed to examine whether family members’ participation in telehealth intensive outpatient (IOP) therapy for mental health disorders in youths and young adults is associated with patient’s treatment engagement. A secondary aim was to assess demographic factors associated with family engagement in treatment.

**Methods:**

Data were collected from intake surveys, discharge outcome surveys, and administrative data for patients who attended a remote IOP for youths and young adults, nationwide. Data included 1487 patients who completed both intake and discharge surveys and either completed or disengaged from treatment between December 2020 and September 2022. Descriptive statistics were used to characterize the sample’s baseline differences in demographics, engagement, and participation in family therapy. Mann-Whitney *U* and chi-square tests were used to explore differences in engagement and treatment completion between patients with and those without family therapy. Binomial regression was used to explore significant demographic predictors of family therapy participation and treatment completion.

**Results:**

Patients with family therapy had significantly better engagement and treatment completion outcomes than clients with no family therapy. Youths and young adults with ≥1 family therapy session were significantly more likely to stay in treatment an average of 2 weeks longer (median 11 weeks vs 9 weeks) and to attend a higher percentage of IOP sessions (median 84.38% vs 75.00%). Patients with family therapy were more likely to complete treatment than clients with no family therapy (608/731, 83.2% vs 445/752, 59.2%; *P*<.001). Different demographic variables were associated with an increased likelihood of participating in family therapy, including younger age (odds ratio 1.3) and identifying as heterosexual (odds ratio 1.4). After controlling for demographic factors, family therapy remained a significant predictor of treatment completion, such that each family therapy session attended was associated with a 1.4-fold increase in the odds of completing treatment (95% CI 1.3-1.4).

**Conclusions:**

Youths and young adults whose families participate in any family therapy have lower dropout, greater length of stay, and higher treatment completion than those whose families do not participate in services in a remote IOP program. The findings of this quality improvement analysis are the first to establish a relationship between participation in family therapy and an increased engagement and retention in remote treatment for youths and young patients in IOP programing. Given the established importance of obtaining an adequate dosage of treatment, bolstering family therapy offerings is another tool that could contribute to the provision of care that better meets the needs of youths, young adults, and their families.

## Introduction

### Overview

Many youths and young adults (23%-63%) who seek mental health therapy drop out before completing the recommended dose of treatment [1]. Youths and young adults who drop out of mental health therapy early have smaller reduction in symptoms and are less likely to maintain improvement at follow-up than youths and young adults who complete treatment [2]. As mental health needs awareness among youths and young adults, with 10% having a diagnoseable mental health illness [3], mental health providers need effective methods for engaging youths and young adults in an adequate treatment dose for symptom improvement.

Family engagement is a uniquely powerful aid for youths and young adult treatment retention and effectiveness. Youths’ treatments that include family members have significantly better outcomes [4-7]. Interviews with caregivers indicate they are often the catalyst for youths to enter and maintain treatment [8], and a systematic review found that friends and family are essential pathway agents in helping youths and young adults to access and stay engaged in treatment [9].

Family members engaged in treatment may be better positioned to support treatment retention and adherence for their children. Research in outpatient, in-person treatments indicates that family engagement leads to lower dropout and greater attendance [10,11]. However, little research has investigated family involvement and retention in telehealth services, despite their rising prevalence [11,12]. This research disparity also exists for intensive services, even as the demand for more intensive services increases. The purpose of this study is to assess whether the family involvement in treatment is associated with treatment completion, attendance, and length of stay for young people in a transdiagnostic remote-intensive outpatient program.

### Treatment Engagement Linked to Positive Outcomes

It is a challenge to engage youths and young adults in an adequate dose of mental health therapy to meet treatment goals. In a meta-analysis of youths’ dropout from psychotherapy, 23%-63% of youths withdrew from treatment before the therapist’s recommendation or before goals were met [1]. High rates of treatment dropout can be seen across multiple diagnoses. For example, in a large study of therapy patients with depression or anxiety, nearly a third dropped out of treatment before completion [13], and more than half of youths participating in trauma treatment did not complete it [2]. Youths who drop out of treatment early are at higher risk for poorer outcomes [13,14]. As patients build skills and awareness throughout the treatment, this allows them to increase their skills and reduce symptoms; patients who drop out early may not reach the same well-being as those who attend a full dose. For example, in a retrospective study of 1850 outpatient therapy patients with depression and anxiety, those who dropped out were more likely to still fall above the clinical cutoff for anxiety or depression, and less likely to have seen significant improvement in their depression or anxiety scores [13]. In a study of youths participating in trauma treatment, those who did not complete treatment had smaller improvements in posttraumatic stress disorder symptoms, anxiety, depression, and behavioral challenges, and were more likely than those who completed treatment to fall in the clinical range at follow-up [2]. Although these studies assessed treatment completion, similar patterns have been seen based on the number of sessions attended. In a study of low-intensity outpatient depression treatments, patients who participated in at least 4 sessions had a substantially higher likelihood of clinically significant improvement, and each additional session attended was associated with a 21% increase in the likelihood of clinically significant improvement [14]. These findings emphasize the need for strategies to retain patients in mental health treatment for a high enough dose to make and maintain clinical improvement.

### Family Participation Increases Outpatient Treatment Engagement

#### Overview

Family participation in therapy, as well as caregivers’ belief in the efficacy of treatment, is associated with increased rates of treatment completion for youths in transdiagnostic reviews of treatment, as well as in studies of sexual abuse and trauma treatment specifically [10,15,16]. This finding indicates that giving family members tools to understand the therapeutic process and its potential efficacy may improve the rates of treatment completion for youths. Caregiver engagement in children’s treatment specifically is a key quality indicator for mental health care for youths [17]. The form of family participation varies by program, commonly including therapy sessions with caregivers and other family members, and sometimes including group family sessions, family-school consultations, or home visits [10]. In regard to mental health treatment in particular, it is common to involve the family; for example, in a review of cognitive behavioral therapy randomized controlled trials for a broad variety of youths’ mental health concerns, 62% of the treatments studied involved a caregiver [4]. Similarly, in a review of millions of youths’ psychotherapy claims, a caregiver was present in 46% of the claims [18], and a review of youths’ mental health services found that caregivers attended 42% of sessions [19]. In a systematic review of child and family mental health treatment, there was a consistent link between family participation and retention in treatment across all 6 related studies [10]. One subsequent study examined a treatment where 1 caregiver participated in every session and found that families where another caregiver also participated were more likely to complete treatment [20].

In addition to the benefits to the youths involved with treatment, family participation in youths’ and young adults’ treatment can also address family needs. In a systematic review of studies on the needs of caregivers for children with mental health concerns, caregivers expressed the desire for informational, socioemotional, and instrumental support [21], many of which can be accomplished through family participation in treatment. This can also address the following common barriers to treatment that caregivers identify: not feeling supported by formal service systems, feeling blamed and ignored by the child’s therapists, and feeling dissatisfied with services [22].

#### Demographic Factors Associated With Family Engagement

Many factors increase the likelihood of a family participating in treatment, or place barriers to their participation. Research has consistently indicated that patient gender and age are associated with different family engagement. In a review of millions of claims for youths’ psychotherapy, boys were more likely than girls to have a family member attend, as were younger youths [17]. These same findings were also highlighted in a review of multiple evidence-based program implementations [19]. Little research has assessed factors, such as patient sexuality and the link to family engagement.

The current research base documents a strong connection between family participation and treatment engagement and success for patients in outpatient therapy. However, this relationship has been rarely explored in settings such as intensive or telehealth therapy.

#### Limited Research on Treatment Engagement and Family Participation in Intensive Treatment

A systematic review of treatment engagement, dosage, and outcomes found that most evidence is from outpatient clinics, with “scarce and inconclusive evidence in clinical samples with chronic and severe mental disorders” [23]. Similarly, limited research has investigated how family factors impact treatment engagement in intensive settings. In a review of eating disorder intensive programs, only 29% of the studies reported on caregiver or family factors [24], and none of these studies were listed as assessing engagement.

Understanding the potential impact family factors and engagement have on youths’ outcomes is crucial, particularly among youths with high-acuity needs who are referred to intensive treatment. Youths may rely on family support during times of heightened symptoms that require intensive treatment, particularly as there are bidirectional relationships between stressful life experiences, family support, and heightened youths’ behavioral symptoms [25]. Intensive programs that include family components have documented significant improvements in youths’ outcomes [26-30], although research has rarely focused on family engagement or family factors as an outcome predictor. Strong outcomes with families engaged in intensive treatment may be linked to higher attendance and completion rates. In 1 preliminary study of a psychiatric inpatient-treatment program for adults, a staff member contacted a family member or support person for 75% of participants, and having any family involvement was significantly associated with attending outpatient appointments [31]. Further research is needed to assess the role of family participation in treatment engagement in intensive settings.

#### Limited Research on Family Participation in Telehealth Treatment

Telehealth mental health services have become increasingly common, particularly following COVID-19. Telehealth services include 2-way, real-time web-based communication between the patient and provider in different locations, delivered by video- or audioconferencing [32]. Half of the adults with serious mental illness received telehealth services in 2020 [33]. Telehealth has been shown to lead to an increased individual engagement, with patients in telehealth-intensive therapy attending more sessions than those in in-person therapy [33-35], and evincing a higher likelihood of completing treatment [33]. In a telehealth treatment model, the transportation barrier [36] is eliminated, and interviews with clinicians indicate that youths can take more responsibility for their own treatment schedule and have reduced barriers in participation [37]. Some youths report that family members were more involved in treatment once sessions transitioned to telehealth due to the pandemic [38].

Many youths are still dependent on family members for payment, insurance, and technical assistance, and caregivers specifically remain the primary motivators of treatment initiation and the curators of the home environment [39]. However, little research has assessed family participation in telehealth treatment, or its connection to treatment engagement.

### Current Quality Improvement Study

The current body of literature indicates that family involvement is crucial to ensuring engagement and retention in common mental health treatments for youths and young adults. However, the impact of family involvement on engagement and retention in an intensive, telehealth context has yet to be examined. As telehealth-delivered mental health care becomes more prevalent, it is important to understand the role that family members play in youths’ retention in a telehealth model. The aim of this quality improvement study was to examine whether participating in family therapy during youths’ and young adults’ telehealth intensive outpatient (IOP) therapy for mental health disorders is associated with patient’s treatment engagement. A secondary aim was to assess demographic factors associated with family engagement in treatment.

## Methods

### Program Overview

Charlie Health provides 100% remote IOP services to youths and young adults nationwide. Patients engage in 3 hours of therapy, 3 days a week. All services take place on the internet. Groups are structured into the following three 50-minute blocks: general process group, evidence-based skills building, and experiential (eg, yoga and movement). Patients are assigned to a cohort based on their presenting issues (eg, dialectical therapy and trauma-focused) and into groups within that cohort based on their age and developmental stage (eg, early, middle, and late adolescent groups; younger and older young adult groups). The patient population that Charlie Health serves is characterized as high clinical acuity. That is, patients typically present with significant clinical depression and anxiety, histories of trauma, and other co-occurring behavioral challenges (eg, justice system involvement, self-harm, and suicidality). In a previous quality improvement analysis of this population, at treatment intake, nearly half (47.1%) of the participants met criteria for nonsuicidal self-injury, nearly three quarters (70.8%) were at risk for suicide, and more than two-thirds (68.1%) reported moderate to severe depression [40]. The average length of stay in treatment is 10-12 weeks and determined on a case-by-case basis.

In addition to 9 hours of group therapy per week, patients participate in optional 1-hour individual or 1-hour family therapy (depending on patient and family willingness to participate) with a masters-level, licensed clinician. Family therapy is offered in 2 formats––with and without the patient present, depending on family preference and presenting needs.

In addition to family therapy sessions, Charlie Health offers robust, drop-in family support programing, provided free of charge to all patient families. Parents are encouraged to continue attending support programing as needed both during treatment and following their youths’ or young adults’ discharge from IOP. A variety of 1-hour support, psychoeducation, and mutual aid groups are running throughout each week for parents and caregivers to attend. Topics covered in the groups include supporting mindful communication, sibling support group, and “IOP Roadmap,” which updates parents on the skills their children are learning each week with tips on how to support them (additional information about Charlie Health IOP programing available in other publications [40]).

### Ethics Approval

This quality improvement research was conducted with approval from the Florida State University Institutional Review Board who deem this type of investigation “nonhuman subjects research” given its primary purpose of program evaluation and quality improvement (STUDY00003364).

Charlie Health conducts routine outcomes monitoring to continuously evaluate clinical programing both across the whole population of patients served, as well as for individual subgroups. This study retrospectively evaluated data from patients whose treatment had ended primarily to inform the improvement of ongoing programing. Patients give authorization for their surveys and recorded clinical data to be used for ongoing program evaluation when they agree to treatment; they are provided this information again each time they are provided a survey, and have the option to skip the full survey and/or any individual questions they choose. The current evaluation was undertaken to better understand the direct impact of participation in family therapy on patient engagement in an effort to continue using patient data to inform refinements in operations that additively improve patient outcomes.

### Inclusion Criteria

Patient cases were included in the analysis that (1) had an intake and discharge survey and (2) either completed treatment or dropped out of treatment (herein referred to as “disengaged”). The latter criterion was chosen based on its congruence with the primary aim of this quality improvement study––to explore the differential impact of family engagement on patient engagement in treatment. Consequently, patients who were discharged because of insurance denial or transfer to a higher or lower level of care were excluded. The former criterium was included because of the way the surveys are designed––patient demographics are collected on different surveys (age on intake; gender and sexual orientation at discharge). Given that patient demographics were used to explore differences in family therapy participation, patient cases were required to have complete demographic information.

### Data Collection Procedures

There were the following 2 sources of data for this evaluation: patient-reported outcomes surveys and administrative data. Outcomes surveys are deployed at patients’ first and last IOP sessions and served as the source of demographic information for this evaluation. When the patient logs into their first IOP session, they are moved to a survey room where an outcomes analyst introduces the survey and distributes an individualized Qualtrics link that has the patient’s account number embedded. In the introduction to the survey, patients are informed that their survey data will be used for 2 purposes, which are as follows:

1. “To collect information that is shared with your primary therapist. This will help you and your primary therapist to come up with some goals for you to work on while at Charlie Health,” and
2. “To know how well Charlie Health is working for everyone. When we look at the data across the organization, we remove all your personal information so that your answers are not linked to you personally.”

There are no forced responses in the survey, allowing patients to provide as much or as little information as they are comfortable sharing.

When the patient completes their survey, they are then moved to patient orientation. At the patient’s last IOP group, they are pulled into the survey room to complete the discharge survey in the same manner. Patients that do not attend their last IOP session are emailed a link to the discharge survey with follow-up reminders. Administrative data (length of stay and discharge type) are recorded through an electronic health record system. At the backend, all administrative data and survey data are linked in 1 data file using the patients’ account number. The data file is then deidentified by removing the account numbers and replacing them with meaningless codes (“study IDs”). All data were uploaded and analyzed using SPSS (version 29; IBM Corp).

### Variables

#### Demographic

Demographic information is collected on the patient-reported outcomes survey and includes age (continuous), gender, and sexual orientation of the patient. Gender response options include male, female, gender-fluid, gender-neutral, gender questioning, genderqueer, nonconforming, and nonbinary. Sexual orientation options include asexual or gray-sexual, bisexual, pansexual, gay, heterosexual or straights, lesbian, queer, and questioning.

#### Treatment Engagement

Treatment engagement was operationalized as patient attendance (the number of scheduled sessions attended), length of stay (the number of weeks of IOP attended), and treatment completion (whether a patient completed treatment or dropped out of treatment).

#### Family Therapy

Family therapy sessions offered by CH therapists can include both family therapy sessions with the patient present (eg, youths and their parents meeting together with a therapist, or a young adult and a partner meeting together with a therapist), or family therapy sessions without the patient present (eg, a therapist meeting with the parents of a youth patient, without the youth present). The determination of the type of family therapy patients engage in, is dependent on patient and family preference. For the purposes of this analysis, both types of family therapy sessions were summed for a total count of family therapy sessions.

### Data Preparation

#### Demographic Variables

Gender was recoded from an 8-level variable to a 3-level variable for parsimony given that many of the subgroups had relatively small sample sizes, that is, male, female, and nonbinary (see Table 1). Similarly, the original 9-level sexual orientation variable was reduced to a 4-level categorical variable, collapsing smaller groups into an “other sexual orientation” category, including pansexual, bisexual, heterosexual, and “other sexual orientation.” The variables transgender (yes/no) and age (continuous) were retained in their original form for the analyses.

**Table 1:** Patient demographics and treatment characteristics (N=1487).

			Patients^a^, n (%)		Completed treatment, n (%)		Attendance rate (%), median		Average weeks, median		Family therapy participation, n (%)
Total sample			1487 (100)		1057 (71.1)		80.7		10.00		731 (49.2)
Missing sample			N/A^b^		0 (0)		0		0		0 (0)
**Gender**											
	Male	698 (46.9)		499 (71.6)		81.3		10.5		322 (46.1)	
	Female	416 (28.0)		298 (71.5)		80.5		10.0		217 (51.4)	
	Nonbinary	313 (21.1)		245 (78.3)		81.7		11.0		172 (55.1)	
	Missing	60 (4.0)		N/A		N/A		N/A		N/A	
**Transgender**											
	No	1122 (18.5)		812 (72.4)		81.1		10.0		553 (49.4)	
	Yes	275 (75.5)		206 (74.9)		82.1		11.0		137 (50.0)	
	Missing	90 (6.1)		N/A		N/A		N/A		N/A	
**Sexual orientation**											
	Heterosexual	509 (34.2)		344 (67.6)		80.9		12.0		262 (51.7)	
	Pansexual	318 (21.4)		240 (75.5)		80.6		10.0		146 (46.1)	
	Bisexual	197 (13.2)		146 (74.1)		81.3		11.0		101 (51.3)	
	Other	356 (23.9)		276 (77.5)		81.8		11.0		172 (48.5)	
	*Missing*	107 (7.2)		N/A		N/A		N/A		N/A	

^a^For 1456 participants, the mean age was 17.01 (SD 1.14, range 11-32) years.

^b^N/A: Not applicable.

#### Family Therapy Participation

Family therapy was operationalized in 2 ways to permit explorations of any family therapy and then the dose-response of each family therapy session. To create the continuous family therapy session variable, 2 continuous variables were summed as following: number of family therapy sessions attended with the patient present and the number of family sessions attended without patient (as both the options offered to patients and families). The summative variable of number of family therapy sessions attended was used to explore the effect of each additional family therapy session on the outcome variable. To compare the effects of any amount of family therapy to no family therapy, a dichotomous variable to identify patients with no family therapy while in treatment (“0”) and clients that had at least one family therapy session (“1”).

### Statistical Analyses

#### Overview

Descriptive statistics were used to characterize the sample in terms of demographics, family therapy participation, average weeks attended, and attendance rate. The relationship between family therapy participation and treatment engagement was explored using a series of Mann-Whitney *U* tests on patients’ length of stay (weeks attended) and attendance rate in group IOP sessions by the family therapy subgroups and within age groups. The decision to use nonparametric tests for these 2 outcomes was determined by inspecting the Shapiro-Wilk test of normality, wherein the assumption of normality was violated (*P<*.001 on both). Binary logistic regression was used to evaluate the effect of demographic factors on likelihood of having family therapy and to explore the relative impact of family therapy on type of discharge, accounting for salient demographic factors.

#### Missing Data

For patient-reported demographics, there was missing data for 1%-7.2% of the sample due to patients skipping individual questions (see Table 1 for missing data by variable). There was less than 5% missing data on all dependent variables used in the analyses (see Table 1). Although there are no established cutoffs about the percentage of missing data acceptable for statistical inferences, generally less than 5%-10% of missing data are unlikely to bias results [41]. In this analysis, missing data were handled through pairwise deletion.

## Results

### Patient Demographics and Treatment Characteristics

The sample for this evaluation included patients that were discharged from treatment between December 7, 2020, and September 27, 2022. Out of a total of 1846 (80.6%) patients with a discharge and intake survey, 1487 met inclusion criteria; 19.4% (359/1846) were discharged for a reason other than completing treatment or dropping out (eg, transfer to a higher or lower level of care).

The average age of the total sample was 17.01 (SD 4.14; 1.4%, 31/1487 did not provide an age). The gender composition was 28% (416/1487) male, 46.9% (698/1487) female, 21% (313/1487) nonbinary (4%, 60/1487 of patients chose not to self-identify); 18.5% (275/1487) identified as transgender. Patients who identified as heterosexual or straight comprised 34.2% (509/1487) of the patient sample, followed by bisexual (21.4%, 318/1487) and pansexual (13.2%, 197/1487); 23.9% (356/1487) choose another sexual orientation option; and 7.2% (107/1487) declined to answer. The average length of stay across the sample was 9.8 (SD 5.2) weeks.

Patient demographics and treatment characteristics are provided in Table 1, including demographics for the overall sample and for the subgroup of participants who participated in family therapy. Demographic differences relating to family therapy participation are explored in more detail in the “Demographic predictors of family therapy” section. Table 2 shows the treatment characteristics by participation in family therapy.

**Table 2:** Treatment characteristics by participation in family therapy.

			Participants, n (%)		Completed treatment, n (%)	Attendance rate (%), median		Average weeks, n
**Family therapy**			1483 (100)		1053 (71)	80.7		10
	No	752 (50.6)		445 (59.2)		75.0	9	
	Yes	731 (49.2)		608 (83.2)		84.4	11	
	Missing	4 (0.3)		N/A^a^		N/A	N/A	

^a^N/A: Not applicable.

### Family Therapy and Treatment Engagement

#### Treatment Completion

A chi-square and binominal logistic regression was used to assess the predictive value of family therapy to treatment completion (vs disengagement). Patients with any family therapy were significantly more likely to complete treatment (83.2%, 608/731) compared to patients without family therapy (445/752, 59.2%; *χ*^2^_1483_=103.7; *P*<.001). The overall binary logistic regression model with number of family therapy sessions as a predictor was significant (*χ*^2^_1483_=160.1; *P*<.001). Each additional family therapy session was associated with 1.3 increase in odds of completing treatment (*P*<.001).

#### Attendance Rate

Patients with family therapy (731/1462) had a significantly higher median attendance (median 84.38%) compared to patients with no family therapy (733/1462; median 75.00%; *U*=206,933; *z*=−7.54; *P*<.001).

#### Length of Stay

Patients whose treatment involved family therapy (730/1479), stayed in treatment for significantly longer amount of time (median 11.0 weeks) compared to patients with no family therapy (749/1479) (median 9.0 weeks; *U*=335,672; *z=*7.6; *P*<.001).

### Demographic Predictors of Family Therapy

Next, a binomial regression was used to assess the relative impact of each of the demographic factors for explaining whether or not patients engaged in family therapy (Table 3). The overall regression model with age, gender, transgender, and sexual orientation as predictors was significant (*χ*^2^_6_=238.5; *P*<.001), and explained 21.7% (Nagelkerke *R*^2^) of the variance in whether or not patients had family therapy. Of the 3 demographic predictors, age and sexual orientation were significant. Each year reduction in patient age was associated with a 1.3 increase in odds of having family therapy (95% CI 0.7-0.8). Patients who identified as “heterosexual” was associated with a 1.4 increase in odds of having family therapy relative to patients who identified as “pansexual” (95% CI 0.5-0.9) and “other sexual orientation” (95% CI 0.5-0.96). No other predictors were significant in predicting family therapy.

**Table 3:** Logistic regression predicting likelihood of family therapy based on age, gender, and sexual orientation^a^.

	β	SE	Wald chi-square	*df*	*P* value	Odds ratio (95% CI)
Age	−.2	0.02	164.7	1	<.001	0.8 (0.7-0.8)
Nonbinary	.3	0.2	2.8	1	.1	1.4 (0.9-1.95)
Female	−.1	0.1	0.2	1	.7	0.9 (0.7-1.2)
Other sexual orientation	−.4	0.2	5.6	1	.02	0.7 (0.5-0.9)
Pansexual	−.4	0.2	5.0	1	.03	0.7 (0.5-0.96)
Bisexual	−.4	0.2	3.5	1	.06	0.7 (0.5-1.0)
Constant	4.2	0.3	159.7	1	<.001	68.1

^a^The reference group for gender is male; the reference group for sexual orientation is heterosexual.

### Family Therapy and Demographic Predictors of Treatment Completion

A binary logistic regression was used to explore the effect of number of family sessions on treatment completion, accounting for relevant demographic factors (Table 4). The logistic regression model was significant (*χ*^2^_7_=152.6; *P*≤.001) and explained 15.5% (Nagelkerke *R*^2^) of the variance in treatment completion. Patients who identified as “pansexual” were 1.7 times more likely (95% CI 1.2-2.4) and “other sexual orientation” patients were 1.8 (95% CI 1.3-2.5) times more likely to complete treatment compared to heterosexual patients. Each additional family therapy session attended was associated with 1.4 times increase in odds of completing treatment (95% CI 1.3-1.4). Age was no longer significant in this model.

**Table 4:** Logistic regression predicting likelihood of completing treatment based on age, gender, sexual orientation^a^, and number of family sessions attended.

	β	SE	Wald chi-square	*df*	*P* value	Odds ratio (95% CI)
Age	.02	0.02	1.69	1	.193	1.02 (0.99-1.05)
Other sexual orientation	.59	0.17	12.3	1	<.001	1.8 (1.3-2.5)
Pansexual	.5	0.2	10.3	1	.001	1.7 (1.2-2.4)
Bisexual	.4	0.2	3.6	1	.06	1.5 (0.99-2.2)
Number of family sessions	.3	0.03	85.3	1	<.001	1.4 (1.3-1.4)
Constant	−.2	0.3	0.5	1	.5	0.8

^a^The reference group for sexual orientation is heterosexual.

## Discussion

### Principal Results

This quality improvement study sought to understand the relationship between family engagement in youths’ IOP therapy and its relationship with youths and young adult engagement, retention, and treatment completion. Across both metrics of treatment engagement and treatment completion, and across demographic factors, patients with family therapy sessions were more engaged and more likely to complete treatment. Youths and young adults with one or more family therapy sessions were significantly more likely to stay in treatment with an average of 2 weeks longer (11.0 weeks vs 9.0 weeks), to attend a higher percentage of IOP sessions (84.38% vs 75.00%), and to complete treatment without leaving early (83.2% vs 59.2% treatment completion rate).

Some demographic variables were associated with increased likelihood of participating in family therapy, including younger age (OR 1.3) and identifying as heterosexual (OR 1.4). Finally, examining the number of family therapy sessions attended and after accounting for age and sexual orientation, the study found that with each additional family therapy session attended, patients were 1.4 times more likely to complete treatment. Several possible rationales could explain these findings. It could be that families who agree to attend family therapy, and who have the resources to arrange to attend, are more likely to encourage their youths to attend and complete treatment and to provide any necessary resources to do so. Alternatively, it could be that families who attend family therapy become more invested in the treatment plan and encourage the youths or young adult to continue the treatment until completion, or that families learn new skills or ways of empathizing with the youths or young adults that impart hope and endurance to complete treatment.

### Comparison With Previous Work

The findings of this analysis reflect trends in the extant research on the positive relationship between family engagement and youths’ engagement and retention in outpatient in-person care [10,11,15,16]. Given the dearth of information currently available on treatment engagement in higher acuity settings [23] and the lack of information on the impact of family participation in treatment on youths’ and young adults’ engagement in care in telehealth or intensive services, one of the greatest strengths of the current investigation was the ability to explore this relationship among a large, diverse, nationwide sample of youths and young adults receiving treatment in a telehealth, intensive environment.

Notably, only 49.3% (731/1482) of patients had any participation in family therapy. This percentage is similar to administrative studies and reviews of outpatient therapy, where caregivers were present in 42%-46% of cases [17,19], and lower than the engagement in more structured randomized controlled trials [4]. This study’s findings that IOP, like other outpatient services, has only half of the patients participating in family therapy suggests that many families either lack interest or face barriers to participating, and that the barriers extend beyond the transportation challenges found in in-person studies.

The finding that younger patients are more likely to have family therapy sessions is consistent with past research in outpatient and in-person settings [17,19]. This study is the first to our knowledge to investigate sexual orientation and parent participation in mental health treatment, presenting a unique opportunity to learn that heterosexual youths and young adults are more likely to have family therapy than youths who identify as pansexual or with “other” sexual orientations. These lower rates of participation in family therapy are concerning, particularly because family support is linked longitudinally to lower mental health distress for LGBT (lesbian, gay, bisexual, transgender) youths as they transition to young adulthood [42]. However, despite this lower likelihood of family therapy, youths who are pansexual or other sexual orientation were more likely to complete treatment. This suggests that there may be unique resilience factors among these groups. Given the key role of community support for psychosocial functioning among members of sexual minorities [43], it may be that finding a community, such as peers of similar identities in an intensive program, is particularly influential for youths with stigmatized identities and lower family participation, leading to greater treatment completion.

Research on the impact of family therapy dosage on treatment engagement is in preliminary stage, and so the finding that youths and young adults who attended additional sessions were more likely to complete treatment adds to the limited literature.

### Limitations

Because this was a quality improvement initiative designed to inform operations decisions at 1 company, key limitations should be noted. First, families of patients at Charlie Health are encouraged to attend family therapy and to participate in their youths’ or young adults’ treatment, but it is ultimately up to each family if they wish to participate or not. Because this is a descriptive analysis, it does not account for existing differences in families that opt to participate in their youths’ or young adults’ care. Future research should seek to better understand what differentiates those families that opt into family therapy and those that do not, and whether there are opportunities to engage families in care outside of family therapy (eg, optional support groups).

At the time of submission of this paper, there was not enough data collected to include a variable assessing additional family programing (eg, free family support groups) in order to evaluate the potential additive impact of other program offerings being available. Future research should assess the types of family programing and the number of sessions attended, to assess the additive impact of different types of family programing dosage on youths’ engagement in treatment. Inclusion of data points that represent all programing offered to families and caregivers will be important in future studies in order to understand the interaction among different levels and types of participation in youths’ and young adults’ care.

Beyond attendance, there are additional measures of family involvement that could be captured in future research. Although attendance is the most common method of providers assessing engagement, some providers also assess the level of participation during the session or completion of out-of-session homework, and there are opportunities to use patient expectations of treatment and understanding of treatment goals [44]. Future research could also extend beyond measuring the impact of family participation on treatment engagement to measure its impact on clinical outcomes, such as changes in symptoms, coping skills, and well-being.

### Implications

It is a challenge to engage youths and young adults in a sufficient dose of therapy to improve and maintain improvement in symptoms. The finding that any family therapy participation is associated with significantly higher patient engagement and treatment completion in remote, intensive services just as it is for in-person outpatient services [10,11,15,16] suggests remote-treatment programs should consider including family therapy as an integral part of program offerings, if they do not do so already. Future research could include controlled comparisons of family therapy in intensive settings for a more rigorous evaluation of their impact on treatment engagement.

As only about half of patients had any participation in family therapy, there is a need to identify and work to address barriers to family participation. Future research should investigate barriers to family therapy engagement and should evaluate participation in and impact of alternative family engagement programs, such as family support groups.

This study further identifies a need for greater attention to family relationships among youths who identify as pansexual or of other sexual orientation, as they are less likely to have family therapy, and to further explore the higher treatment completion among pansexual and other sexual orientation youths and young adults.

### Conclusions

This quality improvement analysis demonstrates that participation in family therapy is linked to increased treatment engagement in remote, intensive treatment for youths and young adults, consistent with previous research on in-person and outpatient treatment [10,15,16]. Each family therapy session attended led to an increase in the chances of treatment completion. Past research suggests that increased treatment engagement is linked to downstream impacts, including the eventual improvement of clinical outcomes and other indicators of recovery important to both youths and young adults and their families [4-7]. As health care systems rise to better meet the needs of youths in mental health crisis, families can be engaged as key informants of treatment process, including for intensive and web-based treatment programs.

## Abbreviations

IOP: intensive outpatient
